# Extracellular Pgk1 or Its Derived Short Peptide Interacted with Membrane-Associated Enolase 2 Receptor: A Potential Therapy for ALS Motor Neuron Degeneration

**DOI:** 10.3390/biom16060893

**Published:** 2026-06-17

**Authors:** Bing-Chang Lee, Juey-Jen Hwang, Huai-Jen Tsai

**Affiliations:** 1 Department of Life Science, Fu Jen Catholic University, New Taipei City 242062, Taiwan; bingchanglee@ntu.edu.tw; 2 Cardiovascular Division, Department of Internal Medicine, Fu Jen Catholic University Hospital, New Taipei City 243004, Taiwan; jueyhwang@ntu.edu.tw

**Keywords:** amyotrophic lateral sclerosis, enolase, motor neuron, neurodegeneration, phosphoglycerate kinase, therapeutic peptide

## Abstract

Amyotrophic lateral sclerosis (ALS) remains an intractable motor neuron (MN) disease with a growing patient population and few effective treatments. Here, we review how extracellular phosphoglycerate kinase 1 (ePgk1) improves neurite outgrowth of MNs (NOMN) and axonal growth, both in vitro and in vivo. Our group first elucidated a novel non-canonical function of ePgk1 as a cross-tissue mediator between nerve and muscle tissues. We then discovered that neural membranous Enolase 2 (Eno2) serves as a receptor of ligand ePgk1 and that ePgk1-Eno2 interaction suppresses the Rac1-GTP/p-Pak1-T423/p-P38-T180/pMK2-T334/p-Limk1-S323 axis, reducing p-Cofilin and promoting NOMN and axonal growth, finally suggesting that the 419th aspartic acid residue of Eno2 mediates this interaction. In a crucial preclinical step, we truncated two short 16-amino-acid derivatives from Pgk1, FD-1/-2, each mediating neuroprotection comparable to that of full-length 417-amino-acid Pgk1 in ALS animal models, in terms of improvements of innervated neuromuscular junction, MN cell bodies, motor performance, and endpoint prolongation. In this context, we also discuss the opposite function driven by Eno1-plasminogen interaction and by Eno2-ePgk1 interaction; the latter results in unfavorable for tumorigenesis. Unlike intracellular Pgk1 roles, ePgk1 is an extracellular factor with anti-angiogenic properties, further positioning ePgk1 and its FD-1/-2 as promising protein/peptide drugs for ALS treatment.

## 1. Amyotrophic Lateral Sclerosis (ALS)

ALS results from the degeneration of motor neurons in the spinal cord, leading to progressive impairment of muscle control, muscle atrophy, and paralysis. Ultimately, atrophy of the diaphragm muscles leads to respiratory failure and death [1,2]. ALS exhibits significant variation in age and location of onset, as well as rate of disease progression. Once symptoms appear in most patients, the disease continuously progresses, with a median survival time of approximately 3 years from symptom onset, primarily owing to respiratory failure. Additionally, about 50% of patients experience symptoms beyond the loss of motor function. Among these, 10–15% are also diagnosed with frontotemporal dementia (FTD), and 35–40% exhibit mild behavioral and/or cognitive changes [3]. Approximately 10% of ALS patients have the familial form (familial ALS, FALS), while the remaining 90% are sporadic (sporadic ALS, SALS), most commonly manifesting around the age of 50 [4]. Currently, 10–15% of ALS cases are associated with known genetic mutations and pathogenic mechanisms, while the remaining majority of cases, particularly sporadic ALS, are still unknown [1,2,4,5]. Over 40 genetic mutations are associated with ALS. The most common pathogenic genes include hexanucleotide expansions in chromosome 9 open reading frame 72 (*C9orf72*), superoxide dismutase 1 (*SOD1*), TAR DNA-binding protein 43 (*TARDBP*), fused in sarcoma (*FUS*), and TANK-binding kinase 1 (*TBK1*) [4,6,7]. Additionally, *NEK1*, optineurin (*OPTN*), sequestosome1 (*SQSTM1*), valosin-containing protein (*VCP*), annexin A11 (*ANXA11*), matrin 3 (*MATR3*) and tubulin alpha 4a (*TUBA4A*) have been increasingly recognized as moderate-penetrance risk genes contributing to ALS susceptibility and pathogenesis [8,9,10]. More recent large-scale genomic and exome sequencing studies have further expanded the ALS genetic landscape by identifying additional candidate risk genes, including *DNAJC7* and *CFAP410* [11,12], as well as newly implicated putative loci, such as *YKT6*, *HTR3C*, *GBGT1*, and *KNTC1*, based on recent large-scale exome analyses [13]. However, the strength of genetic evidence varies across these genes, and some still remain to be further validated. Collectively, these findings highlight the genetic complexity of ALS and suggest that multiple converging molecular pathways, including RNA metabolic dysfunction, impaired protein homeostasis, mitochondrial dysfunction, and neuroinflammation, rather than a single causative mechanism, underlie disease onset and progression. Historically, the clinical diagnosis of ALS has depended on the evaluation of upper motor neuron (UMN) and lower motor neuron (LMN) survival, neuromuscular junction (NMJ) loss, axonal retraction, and persistent unexplained muscle weakness [14]. Despite these criteria, many patients experience a diagnostic delay of up to a year after onset, impacting subsequent treatment. Shefner et al. [15] proposed new, simplified diagnostic criteria for ALS, focusing on identifying UMN and LMN dysfunction in one body region or LMN dysfunction in at least two regions. Consequently, the presence and functionality of motor neurons and NMJs are essential for the timely and accurate diagnosis of ALS.

## 2. The Predicted Growing Population of Patients with ALS

By 2040, the global number of ALS patients worldwide is projected to increase to 376,674, with an average global growth rate of 69% [16]. Around the world, a person is diagnosed with ALS every 90 min [17], while in Taiwan, an average of one new case is reported every 3 days [18]. As medical technology advances and physicians gain more diagnostic experience, more patients are being diagnosed with ALS. It is estimated that nearly 40,000 new cases will be identified globally over the next 20 years [16], representing a rapid increase among rare diseases. Only about 30% of ALS patients have a direct correlation with genetic mutations, indicating that the etiology of 70% of ALS cases remains unknown, making drug development challenging [4,6]. Yet the development of drugs to treat ALS only qualifies as orphan drug development for rare diseases, even though the global market for ALS treatments is expected to be valued at $1.28 billion by 2029 [19].

## 3. Current Treatments for ALS

The drugs noted below are currently available, but no effective treatments for ALS have been developed.

(a) Riluzole is a chemical that blocks glutamate-mediated neural transmission [20]. Daily oral administration can cause side effects, such as dizziness, vertigo, or allergic reactions. Treatment with alcohol consumption may exacerbate liver dysfunction. In the later stages of use, it is often discontinued owing to reduced effectiveness and swallowing difficulties in late-stage ALS patients. In phase 3 clinical trials, Riluzole extended survival by about 3 months or delayed the use of mechanical ventilators in ALS patients [21]. According to the label, it is ineffective for end-stage ALS patients.
(b) Radicave^®^ (Edaravone) is administered as an intravenous injection every two weeks. It contains the active ingredient in Radicut, which acts as a free radical scavenger to protect neurons from oxidative damage [22]. The most common side effects are bruising and gait disturbances. The medication can also cause hives, swelling, shortness of breath, and allergic reactions to sulfites, resulting in substantial side effects. Because oxidative stress has long been associated with ALS, Edaravone was suggested as a possible treatment. It is approved for use in several countries and regions, including Asia, the United States, Canada, and Switzerland. Nevertheless, a study by Witzei et al. [23] indicated that Edaravone’s therapeutic effects are comparable to Riluzole, showing no significant advantage in delaying ALS progression.
(c) QALSODY™ (Tofersen; BIIB067) was approved in April 2023 by the FDA under the accelerated approval pathway. QALSODY uses an antisense oligonucleotide therapy to interfere with the expression of the *SOD1* gene, blocking synthesis of the abnormal SOD1 protein. This treatment slows down respiratory and muscle deterioration, but it does not prolong the lifespan of patients [24]. It is administered via intrathecal injection and is specifically designed to treat ALS patients with *SOD1* gene mutation (~2% of cases).
(d) RELYVRIO™ (AMX0035) is composed of sodium phenylbutyrate, a drug for urea cy- cle disorders, and taurursodiol, a nutritional supplement, in a 3:1 ratio [25]. This medication supports mitochondrial and endoplasmic reticulum function to protect patients and slow neuronal death. This medication is administered daily alongside Riluzole. RELYVRIO was approved by the FDA in September 2022. It can increase survival in ALS patients by 5 months [26], but it carries risks for patients with gastrointestinal diseases and is not suitable for those sensitive to high sodium intake. However, the subsequent phase 3 clinical trial failed to demonstrate a significant clinical benefit compared with placebo, raising uncertainty about its efficacy [27,28]. Following the negative PHOENIX trial, Amylyx initiated voluntary discontinuation/removal of RELYVRIO/ALBRIOZA from the U.S. and Canadian market.
(e) NogoA antibody has been found to inhibit neurite outgrowth and nerve regeneration in studies of nerve injury models [29,30]. This is because NogoA inhibits axonal growth in the peripheral nervous system of injured mammals by activating the Nogo receptor (NgR) [29,30]. Therefore, Ozanezumab, a humanized monoclonal antibody targeting NogoA, has been used to block pathologically upregulated membranous NogoA in the skeletal muscle of ALS patients. Neutralizing NogoA is hypothesized to impede the inhibitory signaling cascade, which is mediated by the Nogo-66 domain and neuronal NgR receptors, which induce p-Cofilin upregulation and subsequent actin cytoskeleton disassembly. Thus, Ozanezumab was originally designed as a therapeutic approach [31]. However, phase 2 clinical trials did not find any delay in disease progression (ALSFRS-R) or differences in survival rates with the use of NogoA antibody. The trial was unsuccessful [32]. The potential reasons are further discussed in paragraph 13 (3).
(f) BrainStorm’s NurOwn stem cell therapy has now gained increasing attention in clinical development [33,34], but it is very costly, and its clinical application is limited by complex manufacturing and administration requirements [34]. As a result, only a few patients would benefit from such targeted therapy [33,34].

In sum, current treatments, such as drugs and stem cell therapies, can only slow disease progression and slightly prolong patient survival from three to six months. These treatments are expensive and have individual side effects and risks, but they do not improve the quality of life or mobility. The development of new drugs would, therefore, be a revolutionary breakthrough for this market.

## 4. Correlation Between Neurodegeneration and Abnormal Muscle Function

Spinal muscular atrophy (SMA), ALS, and spinal-bulbar muscular atrophy (SBMA) are all fatal disorders caused by the degeneration of MNs, ultimately resulting in death. Although the characteristics of MN degeneration in these diseases are well defined, recent studies indicate that dysfunction in other cell types, including muscle cells, also contributes to the process of MN degeneration [35,36,37].

SMA is primarily caused by mutations or deletions in the *SMN1* gene [38] and inadequate expression of *SMN2* [39,40]. Research on an SMA mouse model and patient muscle cells has shown that the expression levels of atrogenes, such as Myogenin, MuRF1, and Atrogin 1, rise in muscle cells during disease onset, leading to denervation [41]. Using trichostatin A to inhibit the production of atrogenes, such as myogenin, in muscle cells before muscle atrophy sets in can decrease the extent of denervation.

SBMA, also known as Kennedy’s disease, is caused by an expanded repeat of CAG sequences (polyglutamine; polyQ) in the first exon of the androgen receptor (AR) gene. Patients suffer from respiratory failure and die from MN death [35]. Although SBMA is considered an MN disease, creatine kinase levels in serum from SBMA patients are 10-fold higher than those in normal individuals, indicating muscle damage. This increase is higher than in other MN or muscle diseases, and it can be detected even before symptom onset [42]. In muscle cells of mice that overexpress the AR protein (AR-HSA), denervation is also observed [43]. While MN cell bodies are relatively preserved, loss of MN axons is common [44]. These results indicate that (1) AR-HSA muscle cells are cytotoxic, leading to the removal of nerves connected to skeletal muscle [43] and (2) MN defects may originate at the distal ends of nerves affected by abnormal muscle cells, eventually leading to the death of MN cell bodies.

ALS has been widely studied, and the causes of MN death can be categorized into autonomous and non-autonomous factors. Autonomous factors involve expression of the mutant protein of interest specifically in MNs, while non-autonomous factors involve the impact of neighboring cell pathology on MNs. The influence of non-autonomous factors on the survival of MNs has become a focus of research. For example, the selective removal of mutant *SOD1* in Schwann cells accelerates the progression of ALS [45]. Astrocytes expressing mutant *SOD1* (*G93A*, *G37R*, *G85R*) selectively result in the secretion of neurotoxic proteins, which cause the death of primary spinal neurons and MN progenitor cells [46]. These findings indicate that non-neuronal cells are important in the development and progression of ALS and imply that the primary origin of ALS dysfunction might not be within neurons themselves. Furthermore, it was found that the expression of mutant *SOD1-G93A* in muscle cells leads to ALS-like muscle atrophy symptoms [47]. The expression of mutant *SOD1-G37R* or *-G93A* in muscle cells causes neuromuscular junction (NMJ) dismantlement and MN loss, which are the early signs of ALS [48]. In *SOD1-G93A* mice, overexpression of IGF-1 specifically in muscle cells was shown to maintain NMJ integrity, delay disease onset, and improve survival rates [49]. Collectively, these findings demonstrate that abnormal muscle cell signaling can contribute to neurodegeneration. However, the specific substances that muscle cells may transmit through the NMJ to influence MN survival remain unknown.

## 5. Overexpression of NogoA in the Skeletal Muscle of Mice Induces ALS Symptoms

ALS is currently recognized as a multifactorial, non-cell autonomous disease. Apart from the loss of MNs, abnormalities in glial cells that support and nourish MNs [50], perisynaptic Schwann cells [51], and skeletal muscle cells [52] also contribute to the development and progression of ALS. Clinical research on ALS has shown that patients with early-stage LMN syndrome (LMNS) who exhibit high levels of NogoA protein in their muscles have a high probability (88%) of their LMNS progressing to ALS [53]. It has also been found that the increase in NogoA expression can be detected three months before the onset of disease. Although the overexpression of NogoA in muscle is considered a significant early marker of ALS progression, it cannot be detected in the blood of ALS patients [54]. This rules out bloodwork for ALS diagnostics. Previous studies using transgenic ALS mice with the *SOD1-G86R* mutation, as well as ALS patients, have confirmed a significant increase in NogoA expression in muscle [55]. When endogenous NogoA is removed from *SOD1-G86R* mice, *G86R/NogoA^−/−^* mice have extended survival times and improved survival rates [56]. Transfection of NogoA-expressing plasmids into the muscles of wild-type (WT) mice also revealed changes in the morphology of the NMJ by reduced size in the area of the postsynaptic side [56]. In ALS patients, the overexpression of NogoA is positively correlated with the loss of motor endplates such that higher NogoA expression indicates more severe disease [57]. Hence, the expression of NogoA in muscle is a crucial marker in the early stages of ALS progression.

## 6. NogoA Overexpression Reduces Pgk1 Secretion and Induces ALS Pathogenesis

To verify whether the proteins secreted from NogoA-overexpressing muscle cells might affect the normal growth of MNs, Lin et al. [58] studied the impact of skeletal muscle cell abnormalities on the development of ALS symptoms. Based on the effects of NogoA in muscle cells, they further theorized that abnormal expression of NogoA in muscle cells might alter cross-tissue mediators between muscle cells and MN cells. As proof of concept, they developed a stable NogoA-expressing Sol8 muscle cell line and demonstrated that overexpression of NogoA in muscle cells leads to notable differences in their secretory proteins compared to normal muscle cells. More specifically, overexpression of NogoA resulted in a significant decrease in some proteins/peptides. After screening, they found that phosphoglycerate kinase 1 (Pgk1) stood out because supplementation of it in the medium improved neurite outgrowth of MN (NOMN) and decreased the protein level of phosphorylated Cofilin (p-Cofilin). Yet Pgk1 is a well-known crucial enzyme in glycolysis and gluconeogenesis. In glycolysis, Pgk1 catalyzes the conversion of 1,3-bisphosphoglycerate to 3-phosphoglycerate, producing one ATP molecule; this step is a reversible reaction involved in gluconeogenesis. However, Lin et al. [58] elucidated a novel non-canonical function of extracellular Pgk1 (ePgk1) as a cross-tissue mediator between nerve and muscle tissues. This non-canonical function of ePgk1 is totally distinct from the well-known function of intracellular Pgk1 in glycolysis, which provides ATP to promote axonal growth.

**Figure 1:**
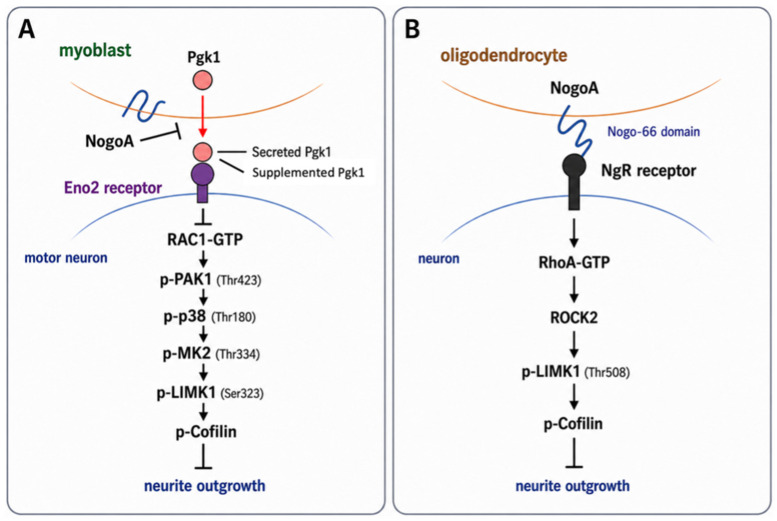
Schematic illustration of the signal pathways driven by ePgk1–Eno2 and NogoA-NgR interactions. (**A**) The signaling pathway triggered by ePgk1–Eno2 interaction across tissues between skeletal muscle and motor neurons as described by Lin et al. [58] and Fu et al. [60]. Either myoblast-secreted Pgk1 or supplementary recombinant Pgk1 interacts with membranous protein Eno2 on motor neurons, suppressing the Rac1-GTP/p-Pak1-T423/p-P38-T180/pMK2-T334/p-Limk1-S323 signaling cascade. Consequently, the level of p-Cofilin, a growth cone collapse marker, is reduced, thereby promoting neurite outgrowth of motor neurons (NOMN). In contrast, NogoA overexpression in muscle leads to a reduction in the secretion of Pgk1 and fails to suppress the Rac1-GTP/p-Pak1/p-P38/p-MK2/p-Limk1 (Ser323) signaling pathway, which results in elevated p-Cofilin levels, thereby suppressing NOMN. (**B**) The signaling pathway triggered by the NogoA-NgR interaction between oligodendrocyte and neurons as reported by Fournier et al. [59]. Being distinct from the non-canonical interaction between Pgk1 and Eno2, the Nogo-66 domain of NogoA interacts with the NgR receptor on neurons through a canonical pathway. This interaction triggers the RhoA/ROCK2/p-LIMK (Thr508) pathway, which increases p-Cofilin to inhibit NOMN. Overall, this figure illustrates the distinct signaling pathways defined by non-canonical Pgk1–Eno2 interaction versus canonical NogoA-NgR interaction.

Lin et al. [58] then went further to prove that adding recombinant Pgk1 to the culture medium also induced NOMN in ALS-modeled MN cells (NSC34-*SOD1* cells) and iPSC-derived neurons from a patient with ALS (*SOD1-G85R*). This led to the discovery of a novel regulatory pathway downregulated by ePgk1, Rac1-GTP/p-Pak1-T423/p-P38-T180/pMK2-T334/p-Limk1-S323/p-Cofilin. This suppression decreases p-Cofilin, causing more active cofilin to be available for the dynamic turnover of the actin cytoskeleton, thereby promoting NOMN. This ePgk1-driven signaling pathway is completely distinct from the ROCK2/p-LIMK-T508/p-Cofilin signaling pathway triggered by the interaction between the Nogo66 domain of NogoA in oligodendrocytes and NgR receptor in neurons, which increases p-Cofilin through increased pLIMK1-T508 by inhibiting actin remodeling, a key mechanism of axonal growth inhibition [59] (Figure 1).

Finally, in vivo analysis in ALS model *SOD1-G93A* transgenic mice showed that injecting Pgk1 significantly reduced or delayed NMJ denervation. The right hind limb injected with Pgk1 displayed better muscle contraction than the left hind limb not injected with ePgk1 [58]. Remarkably, even the addition of a catalytically inactive Pgk1 mutant (Pgk1-T378P) could reduce p-Cofilin-S3 levels, thereby promoting axonal growth. Thus, the NOMN in NSC34 cells induced by exogenous Pgk1 is not a result of increased ATP production through glycolysis. This finding confirms that ePgk1 promotes axonal growth through a mechanism unrelated to glycolysis [58]. This line of evidence confirms that muscle injection of Pgk1 can effectively improve neuromuscular dysfunction in ALS mice, demonstrating that muscle-secreted factor ePgk1 enables the promotion of NOMN and axonal growth, strongly suggesting that ePgk1 could potentially lead to new therapeutics for ALS treatment.

By their 70% genetic homology with humans [61], rapid external development, and transparent embryos, transgenic zebrafish have become an excellent model for neuronal disorders [62,63]. When induced with tetracycline, the transgenic zebrafish line *Tg*(*Zα: TetON-rtn4al*) [64] overexpresses *Rtn4al*, a mammalian orthologue of NogoA, in muscle tissue, leading to muscle atrophy, severe muscle fiber necrosis, and a significant decline in swimming ability, thus closely resembling some of the symptoms of human ALS. When Pgk1 was injected into the dorsal muscles of zebrafish, the number of NMJ colocalization signals increased, indicating that administering Pgk1 by injection could mitigate denervation at the NMJ, alleviating axonal growth defects caused by the overexpression of *Rtn4al* in muscle.

## 7. Cell Membrane Enolase 2 (Eno2) Acts as a Receptor for ePgk1

To address how muscle cell-secreted Pgk1 facilitates cross-tissue communication with nerve cells to promote axonal growth, Fu et al. [60] found that Eno2 (γ-enolase) was the most effective protein in binding to Pgk1-Flag. Additionally, electron microscopy provided direct physical evidence that ePgk1 binds to Eno2 on the cell membrane. In contrast, Pgk1 interacts with neither the Eno1 nor the Eno3 isoform. Meanwhile, overexpression of Pgk1 coupled with overexpression of Eno2 was shown to more strongly suppress the p-P38-T180/p-Limk1-S323/p-Cofilin-S3 signaling axis, reducing the level of p-Cofilin-S3 and highly promoting NOMN in NSC34 cells. An in vivo study also demonstrated that the percentage of MN branching significantly increased in zebrafish embryos after incubation of Pgk1 combined with injection of *eno2* mRNA, whereas no increase was observed in the groups incubated with Pgk1, but combined with either *eno1* mRNA or *eno3* mRNA injection. Moreover, injection of *eno2* morpholino (knockdown) combined with the incubation of Pgk1 confirmed that blocking the endogenous receptor disrupts downstream signaling, impacting MN development. Collectively, these data demonstrate that ePgk1 promotes axonal growth in zebrafish embryos by interacting with Eno2, a member of the enolase protein family, which results in reducing the level of p-Cofilin-S3 through suppressing the p-P38-T180/p-Limk1-S323/p-Cofilin-S3 signaling axis.

In addition to the previously proposed ePgk1-Eno2 interaction, multiple studies have demonstrated that Eno2 possesses neurotrophic and neuroprotective functions. Unlike Eno1 and Eno3, which primarily participate in glycolytic metabolism, Eno2 has been recognized to exhibit additional non-glycolytic neurotrophic properties [65,66]. Previous studies have shown that Eno2 promotes neurite outgrowth, neuronal differentiation, and neuronal survival. Through its interaction with the ligand ePgk1, Eno 2 participates in regulating the PI3K/Akt, MAPK/ERK and RhoA-related signaling pathways [65,67,68]. The C-terminal region of Eno2 has been reported to possess neurotrophic-like activity, and peptide fragments derived from this region can further promote neurite extension and neuronal regeneration [65,66,69].

Several studies have suggested that Eno2 may participate in actin dynamics and growth cone remodeling in association with cytoskeletal reorganization during neurite extension [65,66]. Since neurite outgrowth is highly dependent on actin filament remodeling and growth cone motility, Eno2-enolase-associated cytoskeletal signaling is thought to be closely linked to neurite growth and axonal extension [65,66,68].

**Figure 2:**
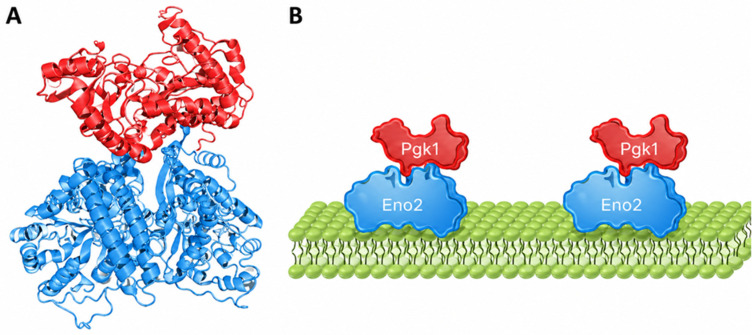
In silico docking prediction and conceptual schematic model illustrating the proposed interaction between extracellular Pgk1 and membrane-associated Eno2. This schematic model was newly generated for the current review article based on previous biochemical interaction studies and ClusPro protein–protein docking prediction. The model is intended to illustrate a proposed conceptual interaction between extracellular Pgk1 and membrane-associated Eno2. (**A**) Structural prediction of Pgk1–Eno2 interaction using the ClusPro protein–protein docking platform. ePgk1 is shown in red, while Eno2 is shown as a dimer in blue. The model illustrates a potential binding interface between ePgk1 and the Eno2 dimer, suggesting structural complementarity that likely supports receptor-dependent functional activity. (**B**) Schematic representation of ePgk1 interacting with membrane-associated Eno2. This conceptual model was developed based on previously published findings reported by Fu et al. [60]. Eno2 (blue) is depicted as a membrane-associated protein embedded in the lipid bilayer (green), whereas Pgk1 (red) is located extracellularly and interacts with Eno2. This model proposes a possible mechanism whereby ePgk1 engages with Eno2 to initiate downstream signaling events.

In summary, ePgk1 inhibits the MAPK p-P38-T180/p-MK2-T334/p-Limk1-S323 pathway, reducing p-Cofilin-S3 and, thereby, enhancing NOMN and axonal growth [58]. Interestingly, zebrafish experimental data also confirmed that the addition of ligand Pgk1, along with the overexpression of receptor Eno2, further enhanced axonal growth. Conversely, knocking down receptor Eno2 promotes the p-P38-T180/p-Limk1-S323 pathway, leading to elevated p-Cofilin-S3 levels and, consequently, inhibiting axonal growth. These findings validate the hypothesis that the interaction between ligand Pgk1 and receptor Eno2 triggers the MAPK/p-P38-T180/p-Limk1-S323/p-Cofilin-S3 signaling pathway to regulate axonal growth. Based on the above evidence, the regulatory ePgk/Eno2/Cofilin pathway is implicated in helping delay and reduce neurotoxicity and apoptosis in MNs. Accordingly, we propose a model in which ePgk1 binds to Eno2 on the neuronal membrane, leading to a reduction in p-P38/p-Limk1/p-Cofilin signaling and promoting axonal growth (Figure 2).

## 8. Eno2 Interacts with ePgk1 to Induce Axonal Growth by One of Key Amino Acid Residues, the 419th Aspartic Acid

**Figure 3:**
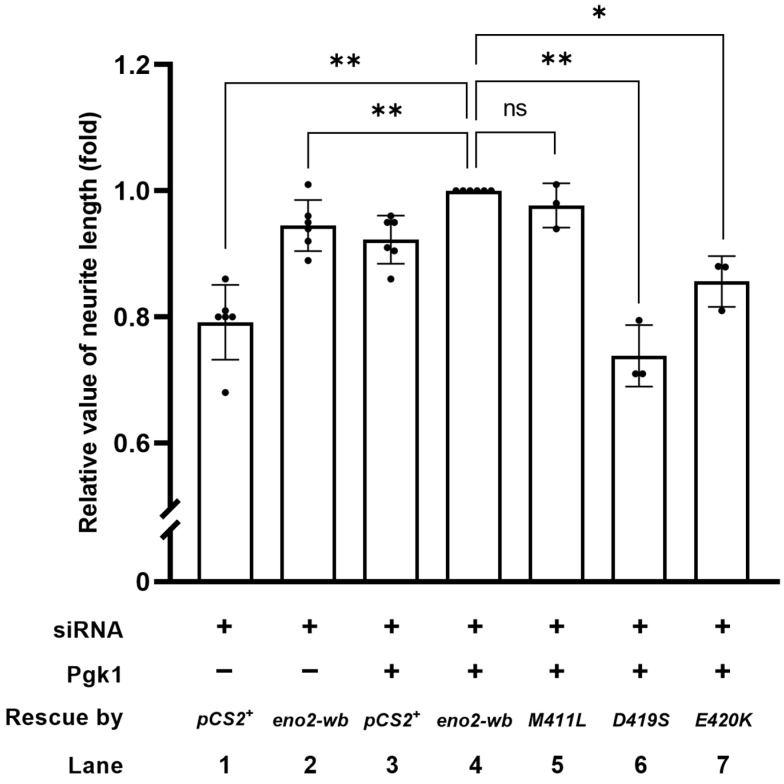
The 419th aspartic acid of Eno2 is a critical amino acid residue required for interaction with ePgk1 to promote the neurite outgrowth in motor neurons (NOMN). A quantitative analysis of the relative length (in fold) of NOMN derived from NSC34 neural cells following knockdown of endogenous Eno2 by siRNA and subsequent rescue by pCS2 (negative control) or wobble-modified Eno2 (*eno2*-wb) (positive control) in the absence (−) or presence (+) of Pgk1. In the absence of recombinant Pgk1, *eno2*-wb could partially restore the reduced length of NOMN caused by *eno2* knockdown (lane 2; n = 6). Upon addition of Pgk1, *eno2*-wb further improved NOMN, indicating a synergistic effect between overexpression of Eno2 and ePgk1 in promoting NOMN (lane 4; n = 6). The eno2-wb-rescued cells supplemented with Pgk1 were defined as the reference group and normalized to 1.0 (lane 4). The ability of each Eno2 mutant (M411L, D419S or E420K) to drive NOMN was individually evaluated in the presence of Pgk1. Compared with *eno2*-wb, the *eno2*-wb-[M411L] mutant retained a comparable ability to drive NOMN (lane 5; n = 3), whereas the *eno2*-wb-[D419S] and *eno2*-wb-[E420K] mutants attenuated the Pgk1-dependent enhancement of NOMN (lanes 6 and 7; n = 3). Each dot represents an independent trial. Data are presented as mean ± SEM (n = 3~6). Statistical analysis was performed using an unpaired two-tailed Student’s *t*-test. Each experimental group was independently compared with the control group. Statistical significance was defined as *p* < 0.05 (*) and *p* < 0.01 (**), while ns indicates no significant difference (this figure was revised and updated from the original figure shown in Lee et al. [70]).

Fu et al. [60] demonstrated that the 325th–417th amino acid domain of ePgk1 binds to the 404th–431st amino acid domain of Eno2, reducing p-Cofilin-S3 and promoting axonal growth. To determine whether any amino acid residue(s) of Eno2 might play major biological roles in NOMN through Eno2-ePgk1 interaction, Lee et al. [70] employed site-directed mutagenesis to generate seven Eno2 mutants, replacing specific residues with the corresponding amino acids of Eno1, as Eno1 cannot interact with ePgk1. For instance, the aspartic acid (D) at position 419 of Eno2 was replaced by serine (S) to generate the Eno2-D419S mutant. They then transfected NSC34 cells with *eno2*-specific siRNA to knock down endogenous Eno2, followed by introduction of a plasmid encoding each mutant to evaluate its ability to rescue reduced NOMN in the presence or absence of recombinant Pgk1. The results are shown in Figure 3 below; in the absence of Pgk1, reduced NOMN in *eno2*-knockdown cells was restored by introduced wobble-modified *eno2* (*eno2*-wb) (lanes 1 vs. 2). However, in the presence of Pgk1, the NOMN of *eno2*-knockdown cells showed a significant increase compared to that of either *eno2*-knockdown cells in the absence of Pgk1 (lanes 1 vs. 3) or *eno2*-knockdown combined with *eno2*-wb transfection in the absence of Pgk1 (lanes 2 vs. 4), suggesting a synergistic enhancement of NOMN mediated by overexpressed interaction between Eno2 and ePgk1.

The synergistic result of NOMN based on *eno2*-wb-cells in the presence of Pgk1 as described above (lane 4) served as a benchmark and was set as 1. Lee et al. [70] then proceeded to quantitatively analyze which mutant(s) might suppress this synergistic effect. The results demonstrated that the *eno2*-wb-[M411L] mutant retained NOMN, similar to that of the benchmark (lanes 4 vs. 5), while the *eno2*-wb-[D419S] and *eno2*-wb-[E420K] mutants significantly impaired it. Specifically, relative to the benchmark reference (lane 4), the eno2-wb-[D419S] mutant reduced NOMN by 0.73-fold (lane 6), whereas the *eno2*-wb-[E420K] mutant showed a 0.85-fold reduction (lane 7). Collectively, the synergistic effect of Eno2-ePgk1 interaction on NOMN was markedly weakened in the Eno2-D419S mutant and was weakened, but not as much, in the Eno2-E420K mutant, suggesting that D419 and E420 of Eno2 may be required amino acid residues for Eno2-ePgk1 interaction in the promotion of NOMN.

Additionally, Lee et al. [70] performed in vivo experiments using the transgenic zebrafish line Tg(mnx1:GFP) with GFP-expressing MNs. In the presence of Pgk1, results showed that the delayed axonal growth in the *eno2*-morpholino knockdown embryos could be restored by injecting *eno2*-wb mRNA. However, when embryos injected with *eno2*-D419S mRNA were also incubated with Pgk1, there was no significant improvement, neither in reducing axonal growth defects nor increasing neuronal branching. Overall, these in vitro and in vivo results clarify the biochemical structural basis for the interaction between ePgk1 and Eno2 and suggest that the 419th aspartic acid residue of Eno2 is permissive for that interaction, since a mutation at this site severely impairs both NOMN (in vitro) and axonal growth (in vivo). These consistent results from in vitro and in vivo systems were only obtained from the D419 mutant, not from the E420 mutant, even though the in vitro experiment described above suggests that both D419 and E420 contribute to Eno2–ePgk1 interaction. Therefore, Lee et al. [70] proposed that the 419th aspartic acid residue of Eno2 is most likely one of the primary determinants crucial for the synergistic enhancement of NOMN mediated by the interaction between Eno2 and ePgk1, whereas the contribution of E420 might be context-dependent or a secondary determinant. Notably, the differential effects caused by various mutants of Eno2 suggest that the charge attraction of amino acids located on the interface between Eno2 and ePgk1 interaction might be a determinant that plays a role in mediating NOMN. However, further studies are needed to identify if additional amino acid residue(s) remaining within the C-terminal domain of Eno2, apart from the 419th aspartic acid, are also involved in Eno2–ePgk1 interaction to regulate axonal and neurite growth.

## 9. Identification of Key Eno Residues That Control the Selective Interaction Between Eno1–Plasminogen and Eno2–ePgk1, Thereby Driving Opposing Biological Functions

Eno1 and Eno2 are very similar proteins, each consisting of 434 amino acids and sharing about 84% sequence identity [71]. Structurally, both proteins include 20 α-helices and 12 β-sheets, and their tertiary structures are almost identical [71]. Despite this high level of structural similarity, the sets of binding partners for Eno1 and Eno2 are different.

**Figure 4:**
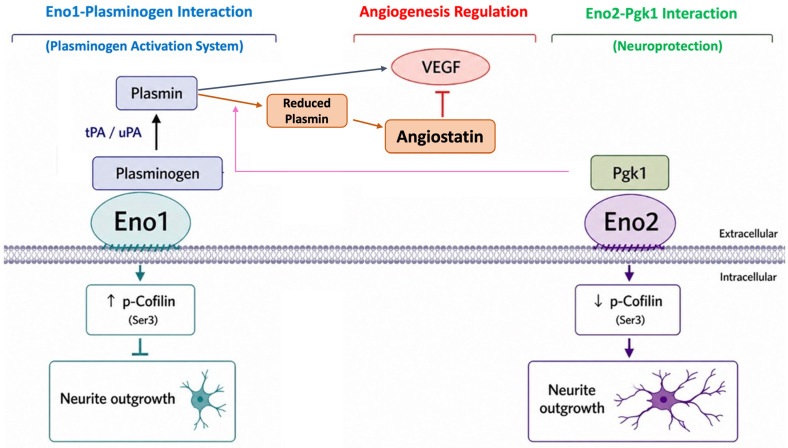
Schematic illustration of the selective interaction between Eno1–Plasminogen and Eno2–ePgk1 to mediate an opposing biological function. This conceptual model was newly generated for the current review article based on previously published findings regarding Eno1–plasminogen interaction, the plasminogen activation system (PAS), and ePgk1–Eno2 signaling as reported by Nakajima et al. [72], Fu et al. [60], and other studies discussed in this review. Eno1 and Eno2 are very similar protein receptors associated with the cell membrane. The Eno1–Plasminogen interaction is illustrated in the left image. Within the PAS, Eno1 binds plasminogen and facilitates its conversion into plasmin in the presence of tissue-type or urokinase-type plasminogen activator (tPA/uPA). This process accelerates fibrin degradation and promotes angiogenesis through extracellular matrix remodeling and the release of growth factors, including VEGF-related signaling. In addition, the interaction between plasminogen and Eno1 is associated with activation of intracellular signaling, leading to increased LIMK1 activity and elevated p-Cofilin levels, which correlates with the inhibition of NOMN. The Eno2-ePgk1 interaction is illustrated in the right image. Extracellular Pgk1 (ePgk1), including endogenously secreted Pgk1 and exogenously supplementary Pgk1, exerts an opposing function driven by the Eno1–Plasminogen interaction. ePgk1 possesses disulfide reductase activity that promotes the generation of angiostatin from plasminogen, thereby suppressing plasmin activity and reducing VEGF expression with a resultant anti-angiogenic effect. Notably, the 419th aspartic acid of Eno2, but not the 419th serine of Eno 1, has high affinity for ePgk1, reducing p-Cofilin and promoting NOMN [60].

Eno1 is well established as a major plasminogen receptor in the brain [72,73]. Binding of plasminogen to Eno1 helps activate the plasminogen activation system (PAS) (Figure 4), which promotes the conversion of plasminogen into plasmin and enhances the breakdown of fibrin [74,75]. This interaction mainly depends on recognizing the C-terminal lysine residues (K420, K422, and K434) of Eno1 [73,76]. Notably, Nakajima et al. [72] found that plasminogen interacts specifically with Eno1, but not with Eno2, suggesting that this specificity mostly comes from the presence of these C-terminal lysine residues.

In contrast, ePgk1 plays an opposing role in this system. Notably, its disulfide reductase activity allows it to produce angiostatin through reduction processes, in turn inhibiting plasmin activity [77] and reducing the production of VEGF [78]. Additionally, Kobayashi et al. [79] showed that VEGF activates LIMK1 and increases p-Cofilin through downstream signaling. This suggests that the ePgk1/Eno2 and plasminogen/Eno1 axes have opposing roles in regulating PAS. Previous study has shown that ePgk1 specifically interacts with Eno2 on the neuronal cell membrane, triggering downstream signaling events and, ultimately, decreasing the p-Cofilin level to promote NOMN [60]. This interaction mainly involves the region spanning amino acids 325th–417th of Pgk1 and the C-terminal region at the residues of 405th–431st of Eno2 [60]. More recently, Lee et al. [70] identified the 419th aspartic acid of mouse Eno2 as a key residue controlling ePgk1-Eno2-mediated MN development since its mutation significantly impedes NOMN in cells, as shown above, and axonal growth in zebrafish.

Taken together, it is plausible that the loop region between the α19 and α20 helices, covering amino acid residues that range from 410th to 426th, is a critical structural component that determines how Eno1 and Eno2 specifically interact with plasminogen and ePgk1, respectively. Therefore, we conclude that there are key amino acid residue(s) among Eno family to control the selective interaction between Eno1–Plasminogen and Eno2–ePgk1, thereby driving opposing isoform-specific biological functions.

## 10. Therapeutic Implications of Intracellular Versus Extracellular Pgk1

Previous studies have linked Pgk1 to malignant tumor phenotypes based on its intracellular roles in metabolism and signaling regulation. Under low oxygen [80], increased Pgk1 promotes glycolysis [81] and triggers epithelial–mesenchymal transition, as well as cell migration and invasion [82], thereby speeding up tumor growth and migration [82]. For example, in oral squamous cell carcinoma, Pgk1 overexpression is closely connected to the activation of AKT signaling, whereas blocking it can significantly reverse malignant features [82]. Additionally, Pgk1 can move to mitochondria and act as a protein kinase to phosphorylate PDHK1, which prevents pyruvate from entering the tricarboxylic acid cycle and further supports the Warburg effect, ultimately encouraging tumor development [80].

Chaytow et al. [83] reported that activating intracellular Pgk1 enzymatic activity with terazosin significantly improves MN survival and function across multiple ALS models by boosting glycolysis and cellular energy metabolism. However, this mechanism aligns closely with the metabolic reprogramming role of Pgk1 in tumor biology, which involves supporting cell survival and growth through increased glycolytic flux. Therefore, while activating intracellular Pgk1 offers neuroprotective benefits, it may also pose risks related to tumor biology, especially in ALS patients with existing cancers or a higher risk of tumor development.

However, other studies have shown that Pgk1 secreted by tumor cells has disulfide reductase activity in the extracellular space [77]. The Pgk1 can further reduce key disulfide bonds within the kringle domains of plasmin to form reduced plasmin [84], causing conformational changes and increasing its vulnerability to subsequent proteolytic cleavage. This process ultimately produces angiostatin from reduced plasmin [77,84] (Figure 4). Angiostatin is an endogenous angiogenesis inhibitor, inhibiting plasmin activity [77] and reducing the secretion of VEGF [78] and interleukin-8, which, in turn, hinders endothelial cell growth, resulting in limiting vascular formation at metastatic sites, thereby suppressing tumor angiogenesis and slowing tumor growth [77], helping to keep tumors in a dormant state [85,86]. Therefore, ePgk1, different from its canonical intracellular counterpart, should be considered an extracellular factor with anti-angiogenic properties rather than a promoter of tumor progression.

Taken together, the expression of intracellular Pgk1 is directly linked to tumor progression, while no concrete evidence currently supports a pro-tumorigenic role of ePgk1. Instead, its main function seems to involve inhibiting angiogenesis through the plasminogen–angiostatin axis. In the nervous system, ePgk1 has also been identified as an extracellular mediator between muscles and MNs, aiding in neuroprotection and the regulation of NOMN and axonal growth [60,87].

## 11. A 16-Amino Acid Short Peptide Derived from Pgk1 Possesses Neuroprotection Equivalent to That of Full-Length ePgk1

Although ePgk1 has potential as a therapeutic candidate for ALS [58], the full-length Pgk1 protein consists of 417 amino acids with a molecular weight of approximately 45 kDa. The relatively large size of the protein would make future chemical modification, industrial-scale production, and manufacturing processes more complex and costly. Therefore, identifying a peptide with a smaller molecular size that retains biological functions comparable to those of full-length Pgk1 would facilitate its future translation into a therapeutic drug.

To achieve this goal, Lin et al. [87] designed a series of domain-mapping experiments to identify the minimal functional domain of Pgk1 that promotes MN growth. Through these analyses, they identified a 16-amino-acid segment spanning residues 345th to 360th of Pgk1, designated as FD-1. Mechanistically, Lin et al. [87] demonstrated that FD-1 inhibits the p-P38-T180/p-Limk1-S323/p-Cofilin-S3 signaling pathway, thereby decreasing the levels of p-Cofilin. This effect was found to encourage actin cytoskeletal remodeling and enhance NOMN, producing neuroprotective effects similar to those of the full-length 417-amino-acid ePgk1 protein. Furthermore, treatment with either FD-1 or its mutant derivative FD-2 (collectively referred to as FD-1/-2) significantly reduced the accumulation of p-Tau-S396 induced by oxidative stress in ALS (*SOD1-G93A*) NSC34 cells. In addition, both peptides alleviated ALS-like pathological features characterized by the mislocalization of TDP-43 from the nucleus to the cytoplasm. These effects were comparable to those observed following treatment with recombinant Pgk1 protein.

In vivo experiments further demonstrated that injecting FD-1/-2 promoted increased motor axon branching in zebrafish embryos. Moreover, these peptides effectively rescued shortened motor axons and impaired locomotor activity seen in zebrafish embryos with *C9orf72* knockdown or mutant *TDP43-G348C* overexpression. In the ALS mouse model, tail-vein administration of FD-1/-2 into *SOD1-G93A* mice significantly delayed denervation at the NMJ in gastrocnemius muscle in a manner comparable to that observed following ePgk1 administration. In addition, ALS mice treated with FD-1/-2 exhibited greater preservation of MNs’ cell bodies in the L5 ventral horn of the spinal cord, increased grip strength, and improved motor performance. Kaplan–Meier survival analysis followed by log-rank testing revealed that treatment with FD-1/-2 significantly prolonged the lifespan of ALS mice (n = 12), even exceeding the survival benefit observed in the Pgk1-treated group. Interestingly, between the two peptides, FD-2 demonstrated superior overall efficacy across multiple pathological and functional indicators compared with FD-1. Collectively, we have developed two Pgk1-based short peptides, FD-1 and FD-2, each consisting of 16 amino acids, that exhibit neuroprotective effects comparable to those of the full-length 417-amino-acid ePgk1 protein. These peptides effectively protect MNs in cellular, zebrafish, and ALS mouse models, suggesting strong potential as peptide-based therapeutics for ALS.

## 12. Multi-Level Screening for Potential FD-Based Short Peptides with Higher Neuroprotective Activity

Lee et al. [88] developed a multi-level screening platform based on a “zebrafish phenotype–first, cellular mechanism validation” strategy. Using the 16-mer peptide (M08/FD-1) derived from residues 345th to 360th of Pgk1 as the benchmark, they systematically compared 17 FD-based mutant peptides, including single-point mutations, multiple mutations, charge-reversal mutations, and length variations, to search for mutant peptides with stronger neuroprotective activity than the benchmark M08/FD-1. In the initial zebrafish phenotypic screening among 17 mutant peptides, the single charge-reversal mutation E358R peptide (M039) exhibited stronger activity in increasing branched CaPMNs compared to that of the benchmark control M08/FD-1-treated group. In vivo analysis further showed that supplementation with M039 showed a more significant recovery of axonal growth defects in *C9orf72*-knockdown ALS-like zebrafish than that of M08. Behavioral analysis also revealed that the improvement in locomotor activity in M039-treated ALS-like zebrafish was notably greater than that observed in zebrafish treated with M08. In cellular models, treatment with M039 increased the NOMN ratio, showing that M039 promotes NOMN growth more effectively than the benchmark peptide M08/FD-1. While mechanistic analysis revealed that both M08 and M039 decreased p-Cofilin expression in ALS-like cells, M039 had the strongest inhibitory effect. Overall, the “zebrafish phenotype–first, cellular mechanism validation” multi-level screening platform, which starts with zebrafish phenotypic screening and moves to cellular validation, enabled quick identification of candidate short peptides with strong neuroprotective and neurite growth-promoting effects for ALS treatment. Further studies are needed to verify whether modified peptide M039 exhibits a higher neuroprotective ability in ALS animal models.

## 13. Translational Benefits of Pgk1-Based Short Peptides

Pgk1, a ubiquitous protein in human cells and a non-artificial molecule, avoids immune reactions by using the patient’s own Pgk1 gene or partial nucleotide sequences and drug-related side effects, potentially shortening the duration of future clinical trials. It is also suitable for both familial (genetic mutation) and sporadic ALS patients, representing personalized medicine. In contrast to the tumorigenic potential of excessive intracellular Pgk1, ePgk1 can inhibit angiogenesis, which does not favor tumorigenesis.

The fundamental neurobiological mechanisms, including the interaction between ligand ePgk1 and receptor Eno2 and the downstream regulatory signaling pathways, have been clearly demonstrated. Additionally, the key amino acid residue(s) responsible for ePgk1-Eno2 binding have been revealed. These findings offer a faster and more accurate approach for future screening and development of small-molecule drugs targeting this pathway. To simplify the manufacturing pipeline, short peptides that retain the MN neuroprotection afforded by the parent Pgk1 are discovered. Compared to the full-length protein, these short peptides significantly reduce the complexity and cost of production, while offering greater flexibility for chemical modification, all contributing to the feasibility of large-scale manufacturing and underscoring the strong translational potential of Pgk1-derived peptides as therapeutic drugs. We highlight the most translational benefits of the Pgk1-based FD short peptides as follows:

(1) FD peptides prolong survival in ALS mice by 12 to 17 days (median). Both peptides provide stable protection to motor neurons, with FD-2 showing greater effectiveness. Based on the cross-species lifespan scaling estimation proposed by Flurkey et al. [89], this survival increase roughly equates to 620 days (about 1 year and 8 months) in humans. This level of effectiveness is highly competitive compared to the current drugs Riluzole and Edaravone, which only extend patient survival by approximately two to three months.
(2) FD peptides increase the preservation of innervated NMJ structures by about 30%. Denervation of NMJs is the common pathological endpoint seen in all forms of ALS, showing that NMJ preservation is a widely relevant therapeutic target. This is especially crucial for most sporadic ALS patients whose disease mechanisms show considerable variance. Consequently, previous treatments aimed at single genes or pathways have repeatedly failed.
(3) Similar to ePgk1, FD peptides inhibit the p-P38-T180/p-Limk1-S323/p-Cofilin-S3 signaling cascade, which decreases p-Cofilin expression and promotes motor neuron growth. This discovery explains why previous therapeutic strategies using the NogoA antibody (Ozanezumab) failed, as they aimed to block the NogoA/NgR pathway to prevent p-Cofilin elevation [32]. Although NogoA antibody inhibits the NogoA/NgR interaction, the key Pgk1/Eno2 interaction and their downstream pathway remain inactive. As a result, the p-Cofilin level cannot be effectively reduced, leading to insufficient promotion of neurite growth, unless exogenous Pgk1 or FD peptides are supplied.
(4) Compared to full-length proteins, antibody-based drugs and gene therapy approaches, these FD short peptides preserve the extracellular neuroprotective mechanism of full-length ePgk1, while providing several other benefits, including smaller size, easier processing and reduced manufacturing costs and quality control, greater flexibility for dose adjustment and molecular modification, and better feasibility for large-scale production and long-term use. FD peptides can also shorten the time required for screening in future drug development.
(5) The sequences of FD peptides derive from the natural human Pgk1 protein, rather than synthetic small-molecule compounds, indicating high biological compatibility and strong translational potential. For rare diseases like ALS, peptide therapeutics also enjoy relatively straightforward regulatory pathways, smaller clinical trial populations, and incentives linked to orphan drug development, further enhancing their potential for clinical application.
(6) FD peptides can alleviate symptoms through extracellular stimulation and improve intracellular conditions without the need for carriers like viruses or liposomes. This enables direct intramuscular or intravenous injections, thus reducing the necessity for complicated invasive procedures.

## 14. Conclusions

ALS remains a devastating neurodegenerative disease with limited therapeutic options and poor clinical outcomes. In this review, we summarize current evidence supporting a novel non-canonical extracellular signaling mechanism mediated by ePgk1-Eno2 interaction that regulates NOMN, preserves the NMJ, and protects MN cell bodies. Unlike the glycolytic function of intracellular Pgk1, ePgk1 plays a distinct cross-tissue neuroprotective role through an ePgk1-Eno2 signaling pathway that modulates cytoskeletal remodeling and p-Cofilin-associated neurite regulation. By identifying Eno2 as the receptor for the ligand ePgk1 and characterizing its key interacting amino acid residues, a potential structural and mechanistic basis for future therapeutic development is established. The development of two 16-amino-acid short peptides, FD-1 and FD-2, further supports the translatability of this signaling concept into Pgk1-based peptide drugs with potential applications in ALS. These innovative short peptides may offer several advantages for therapeutic development, including simpler, low-cost chemical synthesis and purification, potentially lower immunogenicity and toxicity, improved manufacturability, and greater feasibility for large-scale production. Furthermore, these short peptides may exhibit greater target-region specificity by selectively modulating the ePgk1-Eno2 signaling axis, thereby potentially reducing unwanted systemic effects associated with broader intracellular metabolic regulation. Compared with current ALS treatments that mainly provide limited symptomatic relief or modest survival benefits, the ePgk1-Eno2 signaling pathway may represent a first-in-class therapeutic strategy directly targeting MNs. Because ePgk1-Eno2 signaling is biologically distinct from the canonical intracellular metabolic roles of Pgk1, this ePgk1-Eno2 pathway may offer a safety profile that stands in stark contrast with intracellular Pgk1 activation strategies that carry a high risk of tumorigenesis.

Overall, the ePgk1-Eno2 signaling axis and Pgk1-derived peptides may provide a promising foundation for the future development of protein- or peptide-based therapeutics for ALS and other neurodegenerative disorders associated with neurite degeneration and NMJ dysfunction. This study contributes to the development of innovative treatments for rare diseases, offering new hope for patients with ALS.

## 15. Literature Search Strategy

This review was conducted as a narrative and mechanism-focused review, rather than a systematic review or meta-analysis. The relevant literature was searched using PubMed, Web of Science, and Google Scholar databases. The main keywords included “amyotrophic lateral sclerosis”, “Pgk1”, “extracellular Pgk1”, “Enolase 2”, “neurite outgrowth”, “motor neuron degeneration”, “NMJ,” and “neuroprotection.” Priority was given to original research articles and representative studies directly related to extracellular Pgk1 signaling, neuroprotection, and ALS therapeutic strategies. In addition, because this review primarily focuses on extracellular Pgk1-Eno2 signaling, motor neuron neurite outgrowth, NMJ preservation, and ALS-related therapeutic strategies, the selected literature was mainly composed of original studies and representative research articles directly relevant to these topics.

## Data Availability

No new data were generated or analyzed in this study. Data sharing is not applicable to this article.
